# Asking questions changes health-related behavior: an updated systematic review and meta-analysis

**DOI:** 10.1016/j.jclinepi.2020.03.014

**Published:** 2020-07

**Authors:** Lisa M. Miles, Angela M. Rodrigues, Falko F. Sniehotta, David P. French

**Affiliations:** a Manchester Centre for Health Psychology, University of Manchester, Oxford Road, Manchester, M13 9PL, UK; b Department of Psychology, Northumbria University, Northumberland Building, Newcastle upon Tyne, NE1 8ST, UK; c NIHR Policy Research Unit Behavioural Science, Newcastle University, Baddiley Clark Building, Newcastle upon Tyne, NE2 4BN, UK

**Keywords:** Question-behavior effect, Bias, Measurement reactivity, Randomized controlled trial, Behavior

## Abstract

**Objectives:**

The question-behavior effect (QBE) refers to whether asking people questions can result in changes in behavior. Such changes in behavior can lead to bias in trials. This study aims to update a systematic review of randomized controlled trials investigating the QBE, in light of several large preregistered studies being published.

**Study Design and Setting:**

A systematic search for newly published trials covered 2012 to July 2018. Eligible trials randomly allocated participants to measurement vs. non-measurement control conditions or to different forms of measurement. Studies that reported health-related behavior as outcomes were included.

**Results:**

Forty-three studies (33 studies from the original systematic review and 10 new studies) compared measurement vs. no measurement. An overall small effect was found using a random effect model: standardized mean difference = 0.06 (95% CI: 0.02–0.09), *n* = 104,388. Statistical heterogeneity was substantial (*I*^2^ = 54%). In an analysis restricted to studies with a low risk of bias, the QBE remained small but significant. There was positive evidence of publication bias.

**Conclusion:**

This update shows a small but significant QBE in trials with health-related outcomes but with considerable unexplained heterogeneity. Future trials with lower risk of bias are needed, with preregistered protocols and greater attention to blinding.

**What is new?**

**Key findings**
• The QBE appears to be a genuine phenomenon albeit small and inconsistently found.
• Evidence on the QBE has considerable unexplained heterogeneity and is at risk of publication bias.

**What this adds to what was known?**
• Risk of bias is a concern in primary RCTs, but the QBE is still evident in RCTs with a low risk of bias.

**What is the implication and what should change now?**
• Future RCTs need to be pre-registered and require close attention to risk of bias.
• The QBE is a potential source of bias in RCTs with behavioural outcomes.

## 1 Introduction

Existing systematic reviews have supported the idea that measurement can affect behavior [[1], [2], [3], [4], [5]]. Much of this evidence derives from studies where people who were asked to complete a questionnaire showed changes in behavior relative to a control group. This phenomenon is often called the ‘question-behavior effect’ (QBE). The findings of these systematic reviews are consistent: (a) there are overall small effects of asking questions on objective and subjective measures of behavior; (b) there is considerable heterogeneity in effects on behavior across primary studies; (c) many of the primary studies in the reviews have high risk of bias, with a lack of preregistration of protocols as a particular weakness; and (d) publication bias is present in the reviews, but not of sufficient extent to reduce best estimates of effects on behavior to zero. Theoretical explanations of how asking questions can produce changes in people include by increasing awareness of own behavior; providing information about consequences of behavior; or attentional effects through increasing the salience of components of health, behavior, or the link between the two. These explanations suggest that being asked questions may produce increases in health-promoting behaviors [4,5]. The QBE is a specific, well-recognized example of measurement reactivity (MR), which describes the phenomenon where any type of measurement (including objective and subjective measures) can affect the people being measured in terms of cognition, emotion, and behavior [6].

Assessing the strength of evidence for and quantifying the QBE is important because MR may introduce bias in otherwise well-conducted randomized controlled trials (RCTs). Bias may occur because the usual methods of conduct and analysis of trials implicitly assume that the taking of measurements does not affect subsequent outcome measurements, interact with the trial intervention, or that any effects of measurement-taking will be the same in each experimental group and hence are unlikely to bias treatment comparisons. Where any of these implicit assumptions are incorrect, the presence of the QBE is likely to result in incorrect estimates of the intervention effect. The MEasurement Reactions In Trials (MERIT) project has developed Medical Research Council (MRC)/ National Institute for Health Research (NIHR) guidance on minimizing the risk of bias in trials of health care interventions as a result of MR [7]. We report here on an update of an existing systematic review [2] of the QBE on health-related behavior that was conducted to provide an evidence base for the new guidance [7].

An updated evidence base on the QBE is required because many of the trials to date exhibit a high risk of bias [8]. A lack of trials with preregistered protocols is a particular limitation to existing studies [2]. In recent years, some RCTs have been published with a lower risk of bias and preregistered protocols [9,10] including some large ones with null findings [9].

The systematic review by Rodrigues et al. [2] has been selected for updating because it focusses on health contexts, includes only RCTs as the most robust study design for testing the effectiveness of an intervention, and includes a thorough assessment of risk of bias of existing studies [8]. There was a need to update this systematic review, given that the original search for this review was conducted in December 2012.

The objectives of this updated systematic review were to provide an updated estimate of the effect size of the QBE for all RCTs including new studies, to explore several moderators of the QBE, and to assess whether the effect size is robust with regard to risk of bias of included studies and inclusion of studies with/without a preregistered protocol.

## 2 Materials and methods

The protocol for this updated systematic review was published in the PROSPERO database (CRD42018102511).

### 2.1 Inclusion criteria

Trials randomly allocating any type of participant to measurement or non-measurement control conditions or trials in which groups were randomly allocated to different forms of measurement were eligible. Eligible studies reported health-related behavior as outcomes, defined as behavior judged to reduce the risk or severity of diseases or promote health including preparatory behaviors [11]. We included studies with any length of follow-up, although eligible outcomes needed to be assessed at a separate time point to the intervention manipulation measures, that is, studies comparing measures across different formats (e.g., interviews vs. online) were excluded. See Table 1 for PICOS criteria for inclusion and exclusion.

**Table 1:** PICOS criteria for inclusion and exclusion of studies

Parameter	Inclusion criteria	Exclusion criteria
Participants	Any type of participant	
Intervention	Measurement condition: measurement or assessment of cognitions; behavior; or cognitions and behavior; by questionnaire (paper and pencil or online) or interview Alternative intensive measurement condition	Objective measurement (e.g., pedometer, blood pressure monitor)
Comparator	No measurement condition Alternative minimal measurement condition	
Outcomes	Self-reported or objectively assessed health-related behavior	Predictors of behavior (intention and self-efficacy)
Study design	Randomized controlled trials	Non-randomized controlled trials Observational studies

### 2.2 Search strategy

A systematic search of MEDLINE, Cochrane Register of Controlled Trials (CENTRAL), EMBASE, and PsycINFO was conducted from 2012 to July 2018, using the same terms as used in the original systematic review (Supplementary material 1) [2]. In addition, key authors in the research field were invited to provide any additional published literature that fulfilled the inclusion criteria, and a SCOPUS citation search of the original systematic review was conducted.

### 2.3 Study selection and data extraction

**Fig. 1:**
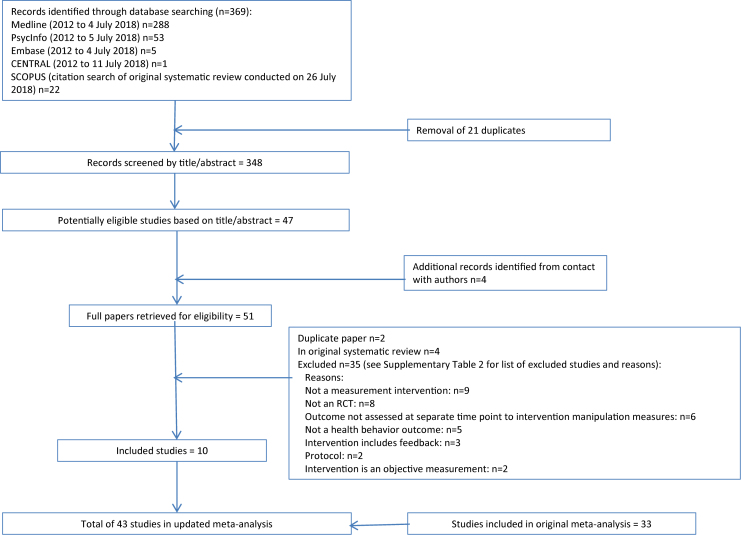
Study selection flow diagram. RCT, randomized controlled trial.

Screening titles and abstracts, and then full papers, for eligibility was completed independently by two reviewers (L.M. and A.R.). Full text was retrieved for 51 papers taking an inclusive approach (see Figure 1); the full paper was scrutinized where the title/abstract was identified by either L.M. or A.R. Each full paper was then assessed independently according to the inclusion criteria (κ = 0.78). For six papers, the reviewers could not decide on inclusion, so consensus was reached after discussion with a third reviewer (D.F.). Data extraction was completed independently by two reviewers (L.M. and A.R.) using an extraction form developed for the original systematic review which covered study and participant characteristics, details of the intervention and control groups, and health behavior outcomes.

### 2.4 Assessment of risk of bias

Risk of bias was appraised independently by two reviewers (L.M. and A.R.) using version 1 of the Cochrane risk of bias tool [12]. Each study was appraised against seven criteria: adequate sequence generation, allocation concealment, blinding (participants, personnel and assessors), incomplete outcome data addressed, and free of selective outcome reporting. The papers were categorized as low, unclear, or high risk of bias and scored 0, 1, or 2, respectively, for each of the seven risk of bias criteria. There was substantial agreement between the two reviewers (κ = 0.78). Overall risk of bias scores were then calculated ranging from 0 to 14; higher scores indicated a greater risk of bias. For two papers, the reviewers could not decide on the risk of bias score for one and two criteria, respectively, so consensus was reached after discussion with a third reviewer (D.F.). Each paper was also assessed for whether there was a preregistered protocol for the study.

### 2.5 Analysis

Meta-analysis of the included studies was conducted using Comprehensive Meta-Analysis (CMA version 3.3.070) software. Dichotomous and continuous outcomes were combined to produce standardized mean differences (SMDs) for all included studies. Details of the analytic strategy for the deriving the SMDs for studies in the original systematic review is published elsewhere [2]; where relevant, the same principles were applied in making decisions for selecting the most intensive measurement condition and/or merging or selecting reported outcomes. The SMDs and key moderator variables for the newly identified studies were added to the original CMA data file to facilitate meta-analysis of the new data set, using a random effects model.

Heterogeneity across studies was assessed using Cochrane's *Q* statistic and *I*^2^ test statistic. Publication bias was examined by a funnel plot (inverse of the standard errors of effect estimates). This was assessed visually to see whether there was evidence of asymmetry. Egger's regression test [12] was used to formally test for the presence of publication bias.

Subgroup analyses were performed to assess the impact of potential prespecified moderators of the QBE: features of participants (student or non-student), content of measurement (cognition, behavior, or both), measurement of attitudes (yes/no), format of measurement (questionnaire or interview), type of health-related behavior (e.g., physical activity, screening), and outcomes (self-report or objective). SMDs for each subgroup were calculated, alongside Cochrane's *Q* statistic and *I*^2^ test statistic, to assess heterogeneity.

To test the robustness of the systematic review findings, sensitivity analyses were conducted to assess whether there were differences in QBE on the basis of risk of bias, presence of a preregistered protocol for the study, or exclusion of an outlying very large study (*n* = 39,538) [9]. A dichotomous variable of high or low risk of bias was generated based on the risk of bias score: below the median (3.5) indicated a low risk and above the median indicated a high risk. SMDs were generated, alongside Cochrane's Q statistic and *I*^2^ test statistic, to assess heterogeneity.

This systematic review update is reported in accordance with the PRISMA guidance [13].

## 3 Results

Ten papers reporting 10 studies (see Table 2) met the inclusion criteria, in addition to the 41 studies (see Supplementary Table 3) that were included in the original review [2]. Data from each of these 10 studies were suitable to add to the meta-analysis (of 33 studies) presented in the original systematic review. A flow diagram of the study selection process is available in Figure 1. The study characteristics and findings of the studies not included in the meta-analysis in the original systematic review have previously been published [2].

**Table 2:** Study characteristics of studies added to the systematic review update

Study ID	Format of measurement	Content of measurement	Comparator	Health-related outcome	Follow-up	Country	Study setting	Population, age, and gender composition	Sample size at follow-up	Funding	Risk of bias score	Protocol preregistration
Barber et al. (2016) [14]	Interview	Interviewed at baseline and follow-up plus weekly survey interviews over 12 mo about pregnancy and contraception use (behavior)	Control interviewed at baseline and follow-up	Sexual behavior (self-report)	12 mo	USA	Community	Female, age 18–20 yr	Measurement condition = 92; no-measurement condition = 94	National Institute of Child Health and Human Development & National Institute on Drug Abuse	9	No
Carey et al. (2015) [15]	Questionnaire	Sexual health survey (cognition and behavior: informational, motivational, and behavioral skills measures, e.g., knowledge, attitudes, negotiation skills), and either general or sexual health DVD (2 measurement groups combined).	General survey and either general or sexual health DVD (2 control groups combined)	Sexual behavior (self-report)	3 mo	USA	Sexual health clinic	44% female, mean age = 28.5 yr	Measurement condition = 410; no-measurement condition = 420	National Institute of Mental Health	9	Yes
Conner et al. (2017) [16]	Questionnaire	Questionnaire with and without sticky note. Cognition: beneficence, intention, and attitude (2 measurement conditions combined)	No contact and demographics (2 control conditions combined)	Vaccination uptake (objective)	4 mo	UK	Community	56.3% female, mean age 75.7 yr	Measurement condition = 3,420; no-measurement condition = 3,425	UK Economic and Social Research Council	4	No
McCambridge et al. (2013) [17]	Questionnaire	Alcohol assessment only (behavior)	No contact control group	Alcohol drinking (self-report)	3 mo	Sweden	Educational institution	51.5% female; 26.5% = 18–20 yr, 56.1% = 21–25 yr, 17.4% ≥ 26 yr	Measurement condition = 2,594; no-measurement condition = 2,669	The Swedish Council For Working Life and Social Research and Wellcome Trust Research Career Development Fellowship	0	Yes
McDermott et al. (2018) [10]	Questionnaire	Standard invitation letter and questionnaire (cognition: theory of planned behavior constructs)	Standard invitation letter	Health check (objective)	6 mo	UK	Community	43.7% female, 52.4% male, 3.9% missing; 83.0% 40–59 yr, 13.1% 60–74 yr, 3.9% missing	Measurement condition = 3,988; no-measurement condition = 4,095	National Institute for Health Research	0	Yes
Meier et al. (2017) [18]	Questionnaire	Alcohol-related questions on frequency and quantity of consumption and a battery of measures assessing behavior and cognition: consequences, normative perceptions, and diagnostic criteria	Control with minimal assessment (daily drinking grid)	Alcohol drinking (self-report)	1 mo	USA	Educational institution	61.7% female, mean age + 19.97 yr	Measurement condition = 65; no-measurement condition = 47	National Institute of Alcohol Abuse and Alcoholism; Psi Chi, the International Honor Society in Psychology; National Institute on Drug Abuse	7	No
O'Carroll et al. (2015) [9]	Questionnaire	Prenotification letter and questionnaire comprising cognition: health locus of control scale, ICK (disgust), perceived benefit, intention, and anticipated regret.	Standard prenotification letter	Screening (objective)	6 mo	UK	Community	51.0% female; 26.6% 50–54 yr, 20.1% 55–60 yr, 16.5% 60–64 yr, 21.0% 65–69 yr and 15.8% 70–74 yr	Measurement condition = 19,934; no-measurement condition = 19,604	Scottish Government, Department of Health, Chief Scientist's Office	1	Yes
O'Carroll et al. (2016) [19]	Questionnaire	Questions on demographics and cognition: affective attitudes, intention, theory of planned behavior constructs, and anticipated regret.	Questions on demographics	Organ donation (other) (objective)	6 mo	UK	Community	56.5% female, mean age = 41.0 yr	Measurement condition = 2,308; no-measurement condition = 2,330	Scottish Government Chief Scientist's Office	1	Yes
Wilding et al. (2018) [20]	Questionnaire	Questions on cognition and behavior: theory of planned behavior constructs	Demographic questions and theory of planned behavior questions focusing on 6 purchasing behaviors	Aggregate health risk/protective behaviors (other) (self-report)	4 wk	World	Community	49.7% female; mean age = 31.8 yr	Measurement condition = 502; no-measurement condition = 520	UK Medical Research Council	5	No
Wood et al. (2014) [21]	Questionnaire	Questions on healthy eating intentions based on cognition: theory of planned behavior constructs	Word jumble and questions on intentions for internet use (2 control groups combined)	Diet (objective)	Immediately after measurement	UK	Educational institution	73.5% female, mean age = 24.5 yr	Measurement condition = 42; no-measurement condition = 85	Not reported	12	No

Of the 10 new studies, six involved community-based adult populations [9,10,14,16,19,20], three studies involved participants from educational institutions [17,18,21], and one study's participants were attendees of a sexually transmitted infection clinic [15]. For nine studies, the measurement intervention involved questionnaires; one study involved interviews [14]. A wide range of health behavior outcomes were covered: sex-related behaviors [14,15], alcohol drinking [17,18], organ donation [19], healthy snack choice [21], colorectal cancer screening [9], health check attendance [10], influenza vaccination attendance [16] and a combined measure of health-related behaviors covering alcohol use, physical activity, diet, and dental health [20]. Of the 10 new studies, the measurement condition for five of the studies involved questions about cognitions [9,10,16,19,21], two involved questions about behavior [14,17] and three involved questions about cognition and behavior [15,20]. Six studies involved questions about attitudes [10,15,16,[19], [20], [21]].

### 3.1 Meta-analysis

**Fig. 2:**
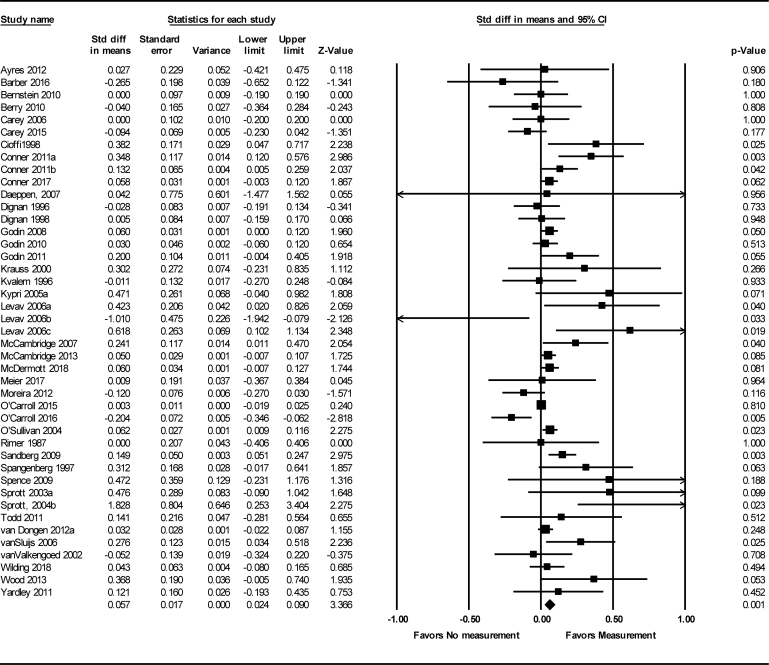
Forest plot of standardized mean differences (SMDs) and 95% confidence intervals (CIs) for health-related behaviors in measurement vs. no-measurement conditions.

For 43 studies (33 studies from the original systematic review and 10 new studies) comparing measurement vs. no-measurement conditions, there was an overall small but significant QBE using a random effect model: SMD = 0.06 (95% CI: 0.02–0.09); *n* = 104,096, see Figure 2. This is slightly smaller than the summary effect size published in the original systematic review (SMD = 0.09, 95% CI: 0.04–0.13, *n* = 37,452). Statistical heterogeneity in the updated meta-analysis is substantial with an *I*^2^ of 54% and a *Q* value of 93.95; df 42, *P* < 0.001. This is an increase in heterogeneity, compared with the original systematic review (*I*^2^:44%, *Q*: 57.39, df: 32, *P*: 0.004).

### 3.2 Sensitivity analyses

Like the original systematic review, the risk of bias among newly identified studies was considerable: scores ranged from 0 to 12; the median score was 4.5 (compared with a range of 0–9 and median 3.0 for the studies in the original systematic review meta-analysis). A breakdown of risk of bias scores by category for each new study is available in Supplementary Table 4. When analyses were restricted to studies with a low risk of bias (score below 3.5), the QBE remains small but significant (SMD = 0.07, 95% CI: 0.04–0.11, *k* = 22, *n* = 90,558) with substantial heterogeneity (*I*^2^ = 63%), see Table 3.

**Table 3:** Standardized mean difference for the question-behavior effect by risk of bias and preregistration of protocol

Sensitivity variable	Measurement group (*n*)	No-measurement group (*n*)	k	*I*^2^	Q	SMD	95% CI
Risk of bias score					1.37		
Below median (3.5)	41,685	48,873	22	63		0.07	0.04 to 0.11
Above median (3.5)	6,854	6,700	21	46		0.03	−0.04 to 0.10
Protocol preregistration					9.04		
Yes	29,566	29,487	6	72		−0.02	−0.07 to 0.04
No	52,291	59,412	37	38		0.09	0.05 to 0.13
O'Carroll et al. (2015) [9]					82.2		
Included	48,539	55,573	43	54		0.06	0.02 to 0.09
Excluded	28,605	35,969	42	50		0.06	0.03 to 0.10

*Abbreviations:* SMD, standardized mean difference; CI, confidence interval.

Half of the newly identified studies had a preregistered protocol [9,10,15,17,19], whereas only one of 41 studies in the original systematic review had a preregistered protocol [22]. A sensitivity analysis of the six studies with a preregistered protocol suggests no evidence of the QBE: SMD = −0.02 (95% CI: −0.07–0.04, *n* = 59,053), with considerable heterogeneity (*I*^2^ = 72%). In the sensitivity analysis of the 37 studies without a preregistered/published protocol, a small positive effect of measurement is demonstrated: SMD = 0.09 (95% CI 0.05–0.13), *I*^2^ = 37%. The sensitivity analysis excluding the large study by O'Carroll et al. [9] did not alter the findings.

### 3.3 Subgroup analyses

Table 4 shows subgroup analyses investigating potential moderators of the QBE. Overall, results are consistent with the original systematic review findings [2]: a larger QBE effect size is reported in students compared to non-students, and the QBE for cognition only measurement conditions (compared with behavior and cognition/behavior conditions) and questionnaire-based measurements (compared with interviews) were significantly different to zero.

**Table 4:** Standardized mean difference for the question-behavior effect by moderator variables

Moderator variable	Measurement group (*n*)	No-measurement group (*n*)	K	*I*^2^	Q	SMD	95% CI
Type of participants					2.65		
Students	3,627	3,836	17	59		0.14	0.03 to 0.26
Non-students	35,253	33,932	26	51		0.05	0.01 to 0.08
Content of measurement					1.62		
Behavior only	3,438	3,502	11	52		0.06	−0.08 to 0.20
Cognition and behavior	1,900	1,991	14	25		0.02	−0.05 to 0.09
Cognition only	33,552	32,275	18	69		0.07	0.03 to 0.11
Measurement of attitudes					0.25		
Yes	21,863	29,267	18	55		0.07	0.02 to 0.11
No	26,607	26,359	25	49		0.05	−0.01 to 0.10
Format of measurement					1.00		
Questionnaires	37,821	36,842	36	59		0.06	0.03 to 0.10
Interviews	969	926	7	0		0.02	−0.07 to 0.10
Type of health-related behavior					21.84		
Blood donation	7,574	6,520	4	33		0.05	−0.00 to 0.10
Diet	166	215	4	65		0.21	−0.26 to 0.68
(Alcohol) drinking	3,856	3,950	8	12		0.03	−0.04 to 0.09
Flossing	81	76	2	0		0.50	0.18 to 0.82
Health check	4,248	4,340	3	81		0.27	−0.09 to 0.62
Other	3,154	3	5	50		−0.03	−0.18 to 0.12
Physical activity	573	598	5	0		0.21	0.08 to 0.34
Screening	24,308	32,236	6	53		0.04	−0.01 to 0.09
Sexual behavior	695	675	4	5		−0.07	−0.19 to 0.05
Vaccination uptake	4,020	4,025	2	4		0.07	0.02 to 0.13
Type of outcome					0.00		
Objective	33,544	32,268	16	66		0.06	0.02 to 0.10
Self-report	5,346	5,500	27	45		0.06	0.00 to 0.12

In terms of type of health-related behavior, the QBEs for flossing and physical activity are still intact as there are no new studies on these outcomes. With the publication of new studies, it was possible to assess vaccination uptake as a separate outcome showing evidence of a small QBE (SMD = 0.07, 95% CI: 0.02–0.13). With the publication of new studies, there is now some suggestion that attitudes and type of outcome could be moderators of the QBE. Studies measuring attitudes showed a QBE (SMD = 0.07, 95% CI: 0.02–0.13) and behavioral outcomes measured objectively were affected by questions (SMD = 0.06, 95% CI: 0.02–0.10).

### 3.4 Publication bias

**Fig. 3:**
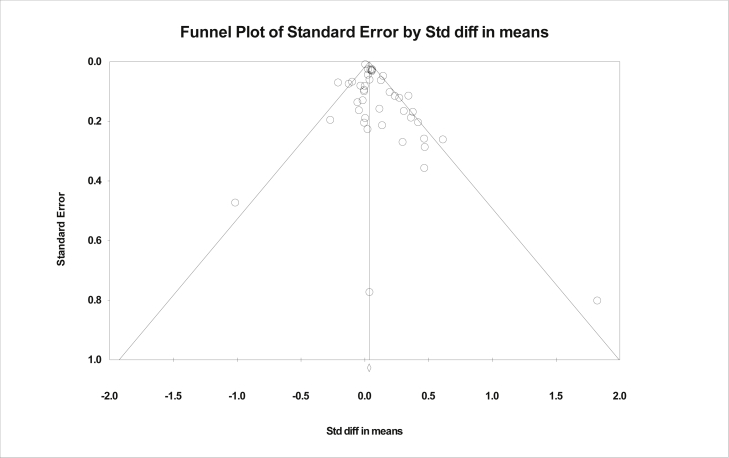
Funnel plot of trials reporting health-related behaviors.

Asymmetry in the funnel plot (see Figure 3) and Egger's regression test (*P* = 0.02) show that there is significant risk of publication bias; this was also identified in the original systematic review [2].

## 4 Discussion

### 4.1 Key findings

Consistent with the original systematic review and other QBE systematic reviews [[3], [4], [5]], the findings of this update show a small but significant QBE in RCTs with health-related outcomes. In an analysis restricted to studies with a low risk of bias, the QBE remained small but significant.

An issue raised in the original systematic review is the possibility that risk of bias of primary studies could produce overestimates of the observed QBE [2,8]. The systematic review update showed that the methodological quality of the included studies was variable, and the risk of bias in the newly identified studies was comparable, both in terms of variability and overall scores. Importantly, the present review showed that the QBE remains intact when restricted to studies with a lower risk of bias. However, the substantial heterogeneity in the sensitivity analysis of studies with a low risk of bias indicates that there is still a lot of unexplained variance, likely due to large variation in studies with respect to content of measurement, types of health-related outcomes, length of follow-up, and characteristics of participants. Findings on potential moderators of the QBE are broadly consistent with existing evidence [4,5].

An important quality criterion raised in the discussion of the original systematic review was whether each included trial had an associated preregistered protocol [2,8]. Only one study in the original systematic review had a protocol preregistered [22], but five of the 10 studies identified in the review update had protocols preregistered [9,10,15,17,19]. The present review showed that the QBE remains intact when new studies with preregistered protocols are included, but a sensitivity analysis restricted to the six studies with a preregistered protocol suggests no evidence of the QBE. This sensitivity analysis showed substantial unexplained heterogeneity. Within this small group of studies, there is large variation in content of measurement and types of health-related outcomes in particular.

Preregistration of trials is a safeguard against publication bias, so it is helpful to consider the results of this sensitivity analysis in light of the funnel plot which detected publication bias. Together, these results suggest that publication bias remains a risk to the evidence base on the QBE. Indeed, previous authors have highlighted publication bias as a particular issue for the QBE literature [2,5,8]. Overall, it is possible that a QBE is undetectable within such a small number of preregistered studies, but nevertheless, this finding suggests there is a risk that the observed QBE is an artifact of publication bias.

Effects sizes of the QBE reported in existing systematic reviews tend to be slightly larger, but these reviews have included non-randomized study designs [4,5] and unpublished data [3] so are not directly comparable. The present authors suggest that the more modest quantification of the QBE in the present review better takes account of the risk of bias and other limitations of existing studies.

### 4.2 Strengths and weaknesses

Systematic reviews specifically follow systematic processes for identifying, selecting, and evaluating relevant studies with a view to minimizing the risk of bias. Particular strengths of this review are the thorough appraisal of risk of bias of included studies, identification and selection of studies for inclusion in duplicate, and exploration of potential sources of heterogeneity. However, the search was limited to the English language and published literature and was not supplemented by handsearching of included studies or topic-related reviews, so it is possible that some studies could have been missed.

### 4.3 Future research and implications

The present review has highlighted the need for further well-designed RCTs with preregistered protocols to facilitate further scrutiny of the impact of publication bias on the QBE literature. All future studies on the QBE need to pay attention to risk of bias, with particular attention to all aspects of blinding (participant, personnel, and assessor). Use of online or automated methods for outcome assessment can help overcome some of these issues. Furthermore, there is a greater need for theorizing about when the QBE is expected and for which groups.

Nevertheless, this systematic review offers empirical support for the idea that measurement can affect the people being measured. There is a need for further primary studies investigating the issue of MR more broadly than the QBE. For example, there is a particular absence of evidence around the potential reactivity of dietary assessments. We also need a greater understanding of when and how the QBE (and MR more broadly) leads to bias in trials. This is an important consideration for trial design. Approaches to minimizing the risk are addressed in new MRC/NIHR guidance on reducing bias from measurement reactions in trials of health care interventions [7].

## 5 Conclusions

Overall, this systematic review update provides evidence of a small but significant QBE in RCTs on health-related outcomes. A greater proportion of the more recent studies have a preregistered protocol and several are large studies. The QBE remains intact when analyses are restricted to studies with a low risk of bias. Although there is a risk that the QBE is an artifact of publication bias, the present review lends support to the conclusion that the QBE is a genuine phenomenon albeit small and inconsistently found.

## CRediT authorship contribution statement

**Lisa M. Miles:** Project administration, Methodology, Investigation, Data curation, Formal analysis, Writing - original draft, Visualization. **Angela M. Rodrigues:** Investigation, Methodology, Data curation, Writing - review & editing. **Falko F. Sniehotta:** Conceptualization, Methodology, Writing - review & editing. **David P. French:** Conceptualization, Methodology, Funding acquisition, Formal analysis, Writing - review & editing, Supervision.

## Supplementary data

Suppl Material 1

Suppl Table 2

Suppl Table 3

Suppl Table 4

Data Profile
