# Supplementary material for: Asking questions changes health-related behavior: an updated systematic review and meta-analysis
Source: J Clin Epidemiol. 2020 Jul;123:59–68. doi: 10.1016/j.jclinepi.2020.03.014 (PMC7308800; doi:10.1016/j.jclinepi.2020.03.014)
Supplement: Suppl Material 1 [file mmc1.docx]

| **Supplementary material 1: Search strategy for Medline (Ovid interface)**  1. randomized controlled trial.pt. | |  |
| --- | --- | --- |
| 2. controlled clinical trial.pt. | |  |
| 3. randomized.ab. | |  |
| 4. placebo.ab. | |  |
| 5. drug therapy.fs. | |  |
| 6. randomly.ab. | |  |
| 7. trial.ab. | |  |
| 8. groups.ab. | |  |
| 9. 1 or 2 or 3 or 4 or 5 or 6 or 7 or 8 | |  |
| 10. exp animals/ not humans.sh. | |  |
| 11. 9 not 10 | |  |
| 12. interview/ | |  |
| 13. Interview, Psychological/ | |  |
| 14. "Surveys and Questionnaires"/ | |  |
| 15. Health Care Surveys/ | |  |
| 16. exp "weights and measures"/ | |  |
| 17. (complet* adj3 (measure* or scale* or interview* or survey* or questionnaire* or test*)).tw. | |  |
| 18. "Outcome Assessment (Health Care)"/ | |  |
| 19. (panel* adj3 survey*).tw. | |  |
| 20. exp mass screening/ | |  |
| 21. ("follow up" adj1 (outcome* or measure* or score* or interview* or assessment*)).tw. | |  |
| 22. (behavio?r* adj4 measure*).ti. | |  |
| 23. 12 or 13 or 14 or 15 or 16 or 17 or 18 or 19 or 20 or 21 or 22 | |  |
| 24. (behavio?r* adj2 measure*).ti. | |  |
| 25. Behavioral Research/ | |  |
| 26. Health Behavior/ | |  |
| 27. exp patient compliance/ | |  |
| 28. exp self examination/ | |  |
| 29. Treatment Refusal/ | |  |
| 30. Feeding Behavior/ | |  |
| 31. FASTING/ | |  |
| 32. Diet/ | |  |
| 33. Food Preferences/ | |  |
| 34. Illness Behavior/ | |  |
| 35. exp reproductive behavior/ | |  |
| 36. Risk Reduction Behavior/ | |  |
| 37. Risk-Taking/ | |  |
| 38. exp sexual behavior/ | |  |
| 39. exp "tobacco use cessation"/ | |  |
| 40. Motor Activity/ | |  |
| 41. Alcohol Drinking/ | |  |
| 42. ALCOHOLISM/ | |  |
| 43. ("physical exercise*" or "physical activit*").tw. | |  |
| 44. (drink* adj1 (alcohol* or pattern* or problem* or addict*)).tw. | |  |
| 45. 24 or 25 or 26 or 27 or 28 or 29 or 30 or 31 or 32 or 33 or 34 or 35 or 36 or 37 or 38 or 39 or 40 or 41 or 42 or 43 or 44 | |  |
| 46. (panel * adj2 conditioning).tw. | |  |
| 47. (pretest* adj2 (response* or effect* or bias* or reactivity)).tw. | |  |
| 48. (test* adj2 (response* or effect* or bias* or reactivity)).tw. | |  |
| 49. (measurement* adj2 (response* or effect* or bias* or reactivity)).tw. | |  |
| 50. (assessment* adj2 (response* or effect* or bias* or reactivity)).tw. | |  |
| 51. (question* adj2 (response* or effect* or bias* or reactivity)).tw. | |  |
| 52. (interview* adj2 (response* or effect* or bias* or reactivity)).tw. | |  |
| 53. (reactiv* adj2 (response* or effect* or bias* or measure*)).tw. | |  |
| 54. "generated validity".tw. | |  |
| 55. mere measur$.tw. | |  |
| 56. "self prophecy".tw. | |  |
| 57. (solomon adj3 (group$ or design$ or trial$ or study or studies)).tw. | |  |
| 58. (solomon adj2 island$).tw. | |  |
| 59. 57 not 58 | |  |
| 60. 46 or 47 or 48 or 49 or 50 or 51 or 52 or 53 or 54 or 55 or 56 or 59 | |  |
|  |  |  |
| 61. 11 and 23 and 45 and 60 |  |  |
