# Supplementary material for: Asking questions changes health-related behavior: an updated systematic review and meta-analysis
Source: J Clin Epidemiol. 2020 Jul;123:59–68. doi: 10.1016/j.jclinepi.2020.03.014 (PMC7308800; doi:10.1016/j.jclinepi.2020.03.014)
Supplement: Suppl Table 2 [file mmc2.docx]

**Supplementary Table 2: a list of 35 excluded studies (newly published) and reasons for exclusion**

| **Reference** | **Reason for exclusion** |
| --- | --- |
| Guillaumie, L., et al. (2012). The impact of self-efficacy and implementation intentions-based interventions on fruit and vegetable intake among adults. Psychology & Health **27**(1): 30-50. | Not a measurement intervention |
| Guillaumie, L., et al. (2013). Self-efficacy and implementation intentions-based interventions on fruit and vegetable intake among adults: impact at 12-month follow-up. Global Health Promotion **20**(2 Suppl): 83-87. | Not a measurement intervention |
| Peltzer, K., et al. (2013). Screening and brief interventions for hazardous and harmful alcohol use among patients with active tuberculosis attending primary public care clinics in South Africa: results from a cluster randomized controlled trial. BMC Public Health **13**: 699. | Not a measurement intervention |
| Pinnock, H., et al. (2013). Effectiveness of telemonitoring integrated into existing clinical services on hospital admission for exacerbation of chronic obstructive pulmonary disease: researcher blind, multicentre, randomised controlled trial. BMJ **347**: f6070. | Not a measurement intervention |
| Hagoel, L., et al. (2016). Harnessing the question-behavior effect to enhance colorectal cancer screening in an mHealth experiment. American Journal of Public Health **106**(11): 1998-2004. | Not a measurement intervention |
| Beaulieu, D. and G. Godin (2012). Staying in school for lunch instead of eating in fast-food restaurants: results of a quasi-experimental study among high-school students. Public Health Nutrition 15(12): 2310-2319 | Not a measurement intervention |
| Belanger-Gravel, A., et al. (2013). The effect of implementation intentions on physical activity among obese older adults: a randomised control study. Psychology & Health 28(2): 217-233. | Not a measurement intervention |
| Voigt, L., et al. (2018). The effect of mere measurement from a cardiovascular examination program on physical activity and sedentary time in an adult population. Journal of Ecology and Environment **42**(1). | Not a measurement intervention |
| Moskowitz, J. M., et al. (2016). Online smoking cessation program for Korean Americans: Randomized trial to test effects of incentives for program completion and interim surveys. Preventive Medicine: An International Journal Devoted to Practice and Theory **86**: 70-76. | Not a measurement intervention |
| Briney, J. S., et al. (2017). Testing the Question-Behavior Effect of Self-Administered Surveys Measuring Youth Drug Use. Journal of Adolescent Health **61**(6): 743-746. | Not a randomised controlled trial |
| Herens, M., et al. (2016). Health-related quality of life, self-efficacy and enjoyment keep the socially vulnerable physically active in community-based physical activity programs: A sequential cohort study. PLoS ONE [Electronic Resource] **11**(2). | Not a randomised controlled trial |
| Gidron, Y., et al. (2015). Effects of psychological inoculation on indirect road hostility and simulated driving. Transportation Research Part F: Traffic Psychology and Behaviour **30**: 153-162. | Not a randomised controlled trial |
| Carey, M. P., et al. (2013). Optimizing the scientific yield from a randomized controlled trial (RCT): evaluating two behavioral interventions and assessment reactivity with a single trial. Contemporary Clinical Trials **36**(1): 135-146. | Not a randomised controlled trial |
| Cho, H., et al. (2015). Discordance of HIV and HSV-2 biomarkers and self-reported sexual behaviour among orphan adolescents in Western Kenya. Sexually Transmitted Infections **91**(4): 260-265. | Not a randomised controlled trial |
| Pratt, N. L., et al. (2015). Commitment questions targeting patients promotes uptake of under-used health services: Findings from a national quality improvement program in Australia. Social Science and Medicine **145**: 1-6. | Not a randomised controlled trial |
| Schneeberg, A., et al. (2014). Knowledge, attitudes, beliefs and behaviours of older adults about pneumococcal immunization, a Public Health Agency of Canada/Canadian Institutes of Health Research Influenza Research Network (PCIRN) investigation. BMC Public Health **14**: 442. | Not a randomised controlled trial |
| Schultz, A., et al. (2012). Usefulness of parental response to questions about adherence to prescribed inhaled corticosteroids in young children. Archives of Disease in Childhood **97**(12): 1092-1096. | Not a randomised controlled trial |
| Anglewicz et al (2013) The Effect of Interview Method on Self-Reported Sexual Behavior and Perceptions of Community Norms in Botswana. AIDS Behav **17**:674–687 | Outcome not assessed at separate time point to intervention manipulation measures |
| Baxter et al (2013) A pilot study of the effects of interview content, retention interval, and grade on accuracy of dietary information from children. Journal of Nutrition Education and Behavior **45**(4): 368-373. | Outcome not assessed at separate time point to intervention manipulation measures |
| Le, L. C. and L. T. H. Vu (2012). Audio computer-assisted self interview compared to traditional interview in an HIV-related behavioral survey in Vietnam. MEDICC Review **14**(4): 26-31. | Outcome not assessed at separate time point to intervention manipulation measures |
| Melson, A. J., et al. (2016). Self-other differences in student drinking norms research: The role of impression management, self-deception, and measurement methodology. Alcoholism: Clinical and Experimental Research **40**(12): 2639-2647. | Outcome not assessed at separate time point to intervention manipulation measures |
| Buu, A., et al. (2017). Assessment methods and schedules for collecting daily process data on substance use related health behaviors: A randomized control study. Drug & Alcohol Dependence **178**: 159-164. | Outcome not assessed at separate time point to intervention manipulation measures |
| Islam, M. M., et al. (2012). The reliability of sensitive information provided by injecting drug users in a clinical setting: clinician-administered versus audio computer-assisted self-interviewing (ACASI). AIDS Care **24**(12): 1496-1503. | Outcome not assessed at separate time point to intervention manipulation measures |
| Cunningham, J. A., et al. (2016). The impact of asking about interest in free nicotine patches on smoker-s stated intent to change: Real effect or artefact of question ordering? Nicotine and Tobacco Research **18**(5): 1215-1217. | Outcome not a health behaviour |
| Moreau, C., et al. (2014). Unplanned or unwanted? A randomized study of national estimates of pregnancy intentions. Fertility & Sterility **102**(6): 1663-1670. | Outcome not a health behaviour |
| O'Carroll, R. E., et al. (2017). If you needed an organ transplant would you have one? The effect of reciprocity priming and mode of delivery on organ donor registration intentions and behaviour. British Journal of Health Psychology **22**(3): 577-588. | Outcome not a health behaviour |
| Giuse, N. B., et al. (2017). Institute of Medicine measures of social and behavioral determinants of health: A feasibility study. American Journal of Preventive Medicine **52**(2): 199-206. | Outcome not a health behaviour |
| Kuhlmann, T., et al. (2016). Using Visual Analogue Scales in eHealth: Non-Response Effects in a Lifestyle Intervention. Journal of Medical Internet Research **18**(6): e126. | Outcome not a health behaviour |
| Parekh, S., et al. (2014). Randomized controlled trial of a computer-tailored multiple health behaviour intervention in general practice: 12-month follow-up results. The International Journal of Behavioral Nutrition and Physical Activity **11**: 41. | Intervention includes feedback |
| Verkooijen, K. T., et al. (2015). The power of regression to the mean: A social norm study revisited. European Journal of Social Psychology **45**(4): 417-425. | Intervention includes feedback |
| Willems, J. I., et al. (2014). Screening for type 2 diabetes in a high-risk population: effects of a negative screening test after 4 years follow-up. Annals of Behavioral Medicine **47**(1): 102-110. | Intervention includes feedback |
| Boffo, M., et al. (2015). Combining cognitive bias modification training with motivational support in alcohol dependent outpatients: study protocol for a randomised controlled trial. Trials **16**: 63. | Protocol paper |
| Donovan, D. M., et al. (2012). Study design to examine the potential role of assessment reactivity in the Screening, Motivational Assessment, Referral, and Treatment in Emergency Departments (SMART-ED) protocol. Addiction Science & Clinical Practice **7**: 16. | Protocol paper |
| Høj, K., et al. (2018). The effect of cardiorespiratory fitness assessment in preventive health checks: A randomised controlled trial. European Journal of Public Health **28**(1): 173-179. | Intervention is an objective measurement |
| Sutton, S., et al. (2014). Does electronic monitoring influence adherence to medication? Randomized controlled trial of measurement reactivity. Annals of Behavioral Medicine **48**(3): 293-299. | Intervention is an objective measurement |
