# Supplementary material for: Asking questions changes health-related behavior: an updated systematic review and meta-analysis
Source: J Clin Epidemiol. 2020 Jul;123:59–68. doi: 10.1016/j.jclinepi.2020.03.014 (PMC7308800; doi:10.1016/j.jclinepi.2020.03.014)
Supplement: Suppl Table 3 [file mmc3.docx]

*Supplementary Table 3: Characteristics of included studies in the original systematic review*

| **Study ID** | **Format of measurement** | **Type of measure** | **Content of measurement** | **Health-related outcome** | **Follow-up** | **Country** | **Study Setting** | **Population age and gender composition** | **Sample size at follow up** | **Risk of bias score**  0 (low risk) –  14 (high risk) |
| --- | --- | --- | --- | --- | --- | --- | --- | --- | --- | --- |
| [**Ayres, et al. (2013)**](#_ENREF_2) | Questionnaire | Dichotomous | Intention, attitudes and anticipated regret | Health plan uptake (objective) | Immediately after measurement | UK | Community | Mean age: 53.4 (71.2 % female) | Measurement condition: 67  No measurement condition: 79 | 0 |
| [**Bernstein et al. (2010)**](#_ENREF_3) | Questionnaire | Continuous | Drinking behavior, other health behaviors, patient health questions and PTSD symptoms | Alcohol use (self-report) | 12 months | USA | Pediatric emergency department | Age  ≤ 17y = 114  ≥18y = 739 | Measurement condition: 209  No measurement condition: 198 | 4 |
| [**Berry and Carson (2010)**](#_ENREF_4) | Questionnaire | Continuous | Behavior and attitude | Physical activity (self-report) | 7-10 days | Canada | University and community | Students sample: mean age 19.7 (73.7% female)  Community sample: mean age 72.0 (75.4% female) | Measurement condition: 117  No measurement condition: 54 | 7 |
| [**Carey, Carey, Maisto, and Henson (2006)**](#_ENREF_7) | Interview | Continuous | Behavior | Alcohol use (self-report) | 1, 6 and 12 months | USA | University | Mean age: 19.2 (65% female) | Measurement condition: 197  No measurement condition: 197 | 8 |
| [**Cherpitel et al. (2010)**](#_ENREF_10) | Questionnaire | Continuous | Behavior | Alcohol use (self-report) | 12 months | Poland | Emergency Department | 39% <30 years (16% female) | Screened only: 87  Assessed: 97 | 3 |
| [**Cioffi and Garner (1998)**](#_ENREF_11) | Questionnaire | Dichotomous | Cognitions only | Blood donation behavior (objective) | 1-week | USA | University | Not provided | Measurement condition: 277  No measurement condition: 370 | 3 |
| [**Clifford, Maisto, and Davis (2007)**](#_ENREF_12) | Interview | Continuous | Behavior | Alcohol use (self-report) | 6 and 12 months | USA | Treatment Centre for alcohol and other drugs abuse | Mean age: 40.01 (37% female) | Intensive assessment: 59  Least intensive assessment: 62 | 3 |
| [**Conner, et al. (2011)**](#_ENREF_13)**a** | Questionnaire | Dichotomous | Theory Planned Behavior cognitions | Health check attendance (objective) | 4 months | England | GP practice | Mean age: 36.4 (52.3% female) | Measurement condition: 199  No measurement condition: 185 | 0 |
| [**Conner, et al. (2011)**](#_ENREF_13)**b** | Questionnaire | Dichotomous | Theory Planned Behavior cognitions | Vaccination uptake (objective) | 2 months | Canada | Public hospital | Mean age: 38.1 (83.4% female) | Measurement condition: 600  No measurement condition: 600 | 2 |
| [**Daeppen et al. (2007)**](#_ENREF_14)**^^[[1]](#footnote-1)^^** | Interview | Dichotomous | Behavior | % of hazardous drinkers (self-report) | 12 months | Switzerland | Emergency department | Mean age: 36.7 (21.8% female) | Measurement condition: 277  No measurement condition: 257 | 3 |
| [**Dignan et al. (1996)**](#_ENREF_18) | Interview | Dichotomous | Knowledge, intentions and behavior | Pap smear screening attendance (self-report) | 12 months | USA | Tribal community: Cherokee Indian | 63.8% <45 years (100% female) | Measurement condition: 448  No measurement condition: 367 | 7 |
| [**Dignan et al. (1998)**](#_ENREF_19) | Interview | Dichotomous | Knowledge, intention and behavior | Pap smear screening attendance (self-report) | 12 months | USA | Tribal community: Lumbee Native American | Mean age: 42.4 (100% female) | Measurement condition: 413  No measurement condition: 426 | 8 |
| [**Godin, et al. (2008)**](#_ENREF_28) | Questionnaire | Continuous | Theory Planned Behavior cognitions | Blood donation behavior (objective) | 6 and 12 months | Canada | Blood Donors agency | Mean age control: 43.8 (38.7% female)  Mean age measurement: 44.7 (38.3% female) | Measurement condition: 2900  No measurement condition: 1772 | 1 |
| [**Godin, et al. (2010)**](#_ENREF_27)**^^[[2]](#footnote-2)^^** | Questionnaire | Continuous | Anticipated regret and intention | Blood donation behavior (objective) | 6 and 12 months | Canada | Blood Donors agency | Mean age: 30.4 (53 % female) | Measurement condition: 879  No measurement condition: 888 | 2 |
| [**Godin, Bélanger-Gravel, Amireault, Vohl, and Pérusse (2011)**](#_ENREF_23) | Interview | Continuous | Theory Planned Behavior cognitions, anticipated regret, moral and descriptive norms, self-efficacy, facilitating factors and positive feelings | Physical activity (self-report) | 3 months | Canada | Quebec city community | Mean age: 40.2 (47 % female) | Measurement condition: 194  No measurement condition: 180 | 2 |
| [**Krauss et al. (2000)**](#_ENREF_35) | Questionnaire | Dichotomous | Knowledge, perceived partner risk, behavior | Safe sex Index (self-report) | 7 weeks | USA | Community: public spaces | Mean age: 36.7 (100 % female) | Measurement condition: 45  No measurement condition: 28 | 2 |
| [**Kvalem, et al. (1996)**](#_ENREF_36) | Questionnaire | Dichotomous | Behavior | Condom use (self-report) | 6 and 12 months | Norway | High school | 16-20 years (50 % female) | Measurement condition: 148  No measurement condition: 133 | 9 |
| [**Kypri, Langley, Saunders, and Cashell-Smith (2006)**](#_ENREF_37) | Questionnaire | Continuous | Behavior | Alcohol use (self-report) | 6 and 12 months | New Zealand | Primary Health-care clinic | Mean age control: 20.1; Mean age measurement: 20.3 (52.2 % female) | Measurement condition: 126  No measurement condition: 126 | 0 |
| [**Kypri and McAnally (2005)**](#_ENREF_38)**^^[[3]](#footnote-3)^^** | Questionnaire | Dichotomous | Behavior | Fruit and veg consumption, alcohol consumption, and physical activity frequency (self-report) | 6 weeks | New Zealand | University primary Health-care clinic | Mean age: 20.2 (49 % female) | Measurement condition: 64  No measurement condition: 60 | 2 |
| [**Levav and Fitzsimons (2006)**](#_ENREF_39)**a** | Questionnaire | Continuous | Intention to floss | Flossing (self-report) | 2-weeks | USA | University | Not provided | Measurement condition: 51  No measurement condition: 46 | 6 |
| [**Levav and Fitzsimons (2006)**](#_ENREF_39)**b** | Questionnaire | Dichotomous | Behavior | Choice of low or high fat snack (self-report) | Immediately after pre-test | USA | University | Not provided | Measurement condition: 25  No measurement condition: 23 | 4 |
| [**Levav and Fitzsimons (2006)**](#_ENREF_39)**c** | Questionnaire | Continuous | Intention to floss | Flossing (self-report) | 1-week | USA | University | Not provided | Measurement condition: 30  No measurement condition: 30 | 8 |
| **(**[**McCambridge & Day, 2008**](#_ENREF_42)**)** | Questionnaire | Continuous | Questionnaire (General Health questionnaire- GHQ, history of trauma scale – HTS, and alcohol use - AUDIT) | Alcohol use – AUDIT (self-report) | 2-3 months | England | University | Mean age control: 20.7 (66 % female); Mean age measurement: 20.6 (67 % female) | Measurement condition: 156  No measurement condition: 144 | 0 |
| [**Moreira, Oskrochi, and Foxcroft (2012)**](#_ENREF_48) | Questionnaire | Continuous | Behavior, behavior-related problems, perceived norms, positive expectancies) | Alcohol use (self-report) | 6 and 12 months | UK | University | 58.5% 17-19 years (61 % female) | Measurement condition: 369  No measurement condition: 332 | 4 |
| [**O’ Sullivan, Orbell, Rakow, and Parker (2004)**](#_ENREF_51) | Questionnaire | Dichotomous | Perceptions of susceptibility and severity of colorectal cancer and attitudes and personal beliefs | Colorectal cancer screening uptake  (objective) | 6-weeks | UK | Community | Age between 50 and 69 years | Measurement condition: 1944  No measurement condition: 10,413 | 0 |
| [**Rimer et al. (1987)**](#_ENREF_54) | Interview | Dichotomous | Behavior and disease-related information, knowledge and concerns about pain regimens, perceived personal control and anxiety | Medication regimens adherence (self-report) | 4 weeks | USA | Hospitals | Age: 53.9% more than 60y  (44.3 % female) | 230 participants | 7 |
| [**Sandberg and Conner (2011)**](#_ENREF_56) | Questionnaire | Continuous | Theory Planned Behavior cognitions | Physical activity (objective) | 2-weeks | UK | University | Mean age: 19.7  (62.0 % female) | TPB only: 192  TPB + regret: 384 | 2 |
| [**Sandberg and Conner (2009)**](#_ENREF_55) | Questionnaire | Dichotomous | Theory Planned Behavior cognitions, anticipated regret | Cervical screening attendance (objective) | 4 months | England | Central Agency responsible for cervical screening | Mean age: 39.1 (100 % female) | Measurement condition: 1426  No measurement condition: 1277 | 2 |
| [**Spangenberg (1997)**](#_ENREF_59) | Questionnaire | Continuous | Behavior | Health club attendance (objective) | 10 days and 6 months attendance | USA | Health club | Not provided | Measurement condition: 73  No measurement condition: 69 | 3 |
| [**Spence, et al. (2009)**](#_ENREF_61) | Questionnaire | Continuous | Behavior, illness perceptions, self-efficacy, intention | Walking behavior (self-report) | 1 week | Canada | University | 95% <30 years (100 % female) | Measurement condition: 15  No measurement condition: 16 | 5 |
| [**Sprott, Smith, Spangenberg, and Freson (2004)**](#_ENREF_62)**b** | Questionnaire | Dichotomous | Behavior | Health and fitness assessment attendance (self-report) | Immediately after pre-test | USA | University | Not provided | Measurement condition: 61  No measurement condition: 60 | 4 |
| [**Sprott, Spangenberg, and Fisher (2003)**](#_ENREF_63)**a** | Questionnaire | Dichotomous | Behavior | Choice of low-fat or higher fat snack (self-report) | Immediately after pre-test | USA | University | Age not provided (100 % female) | Measurement condition: 36  No measurement condition: 44 | 4 |
| [**Todd and Mullan (2011)**](#_ENREF_65) | Questionnaire | Continuous | Behavior, prototypes and Theory Planned Behavior cognitions, | Alcohol use (self-report) | 2 weeks | Australia | University | Mean age: 19 (100 % female) | Measurement condition: 44  No measurement condition: 42 | 3 |
| [**van Dongen, et al. (2012)**](#_ENREF_66) | Questionnaire | Dichotomous | Intention, attitudes (affective and cognitive), subjective, descriptive and moral norms, self-efficacy and role identity | Blood donation behavior (objective) | 6 months | The Netherlands | Blood Donors agency: new donors | Mean age: 33.4 (67 % female) | Measurement condition: 3518  No measurement condition: 3490 | 2 |
| [**van Sluijs, van Poppel, Twisk, and van Mechelen (2006)**](#_ENREF_67) | Questionnaire and accelerometers (without display) | Dichotomous | Behavior and barriers to PA, knowledge, health process of change, social support and self-efficacy | Physical activity  (self-report) | 6 months | The Netherlands | GP practices | Mean age: 55.7 (54% female) | Measurement condition: 155  No measurement condition: 172 | 3 |
| [**van Valkengoed, Morré, Meijer, van den Brule, and Boeke (2002)**](#_ENREF_68) | Questionnaire | Dichotomous | Behavior | Chlamydia screening attendance (objective) | Not provided | Netherlands | Primary care practice | 15-40 years (63.2% female) | Measurement condition: 143  No measurement condition: 149 | 3 |
| [**Walters, Vader, Harris, and Jouriles (2009)**](#_ENREF_69) | Questionnaire | Continuous | Behavior, readiness to change, normative beliefs | Peak blood alcohol concentration (self-report) | 12 months | USA | University | Mean age: 19.8 (66 % female) | Intensive assessment: 63  Least intensive assessment: 66 | 1 |
| [**Yardley, Miller, Schlotz, and Little (2011)**](#_ENREF_70) | Questionnaire | Continuous | Theory Planned Behavior cognitions, perceived risk of infection | Hand washing (self-report) | 4 weeks | England | GP practices | Mean age: 49.8 (64 % female) | Measurement condition: 77  No measurement condition: 80 | 4 |
| **Studies excluded from meta-analysis** | | | | | | | | | |  |
| [**Kalichman, et al. (1997)**](#_ENREF_32) | Interview and questionnaire | Continuous | Behavior | Sexual risk behaviors (self-report) | 2 weeks | USA | Community: African American | Mean age: 34.0 (100 % female) | 158 participants |  |
| [**Knaus and Austin (1999)**](#_ENREF_33) | Questionnaire | -- | Perceptions, self-efficacy, behavior | Sexual risky behavior Index (self-report) | 8 weeks | USA | University | Mean age: 19.41 (54 % female) | 237 participants |  |
| [**Knaus, et al. (2000)**](#_ENREF_34) | Questionnaire | -- | Behavior | Safe sex behaviors Index (self-report) | 7-8 weeks | USA | University | Mean age: 19 (53.9 % female) | Measurement condition: 47  No measurement condition: 61 |  |

1. Revman could not compute an effect size for this study as counts and events were equal in both groups. For this reason a value was removed in events for each group. [↑](#footnote-ref-1)
2. For this study, groups assessing implementation intentions were not included in the analyses. [↑](#footnote-ref-2)
3. Outcomes were merged to produce a single health-related outcome. [↑](#footnote-ref-3)
