# Supplementary material for: Asking questions changes health-related behavior: an updated systematic review and meta-analysis
Source: J Clin Epidemiol. 2020 Jul;123:59–68. doi: 10.1016/j.jclinepi.2020.03.014 (PMC7308800; doi:10.1016/j.jclinepi.2020.03.014)
Supplement: Suppl Table 4 [file mmc4.docx]

*Supplementary Table 4: Risk of bias scores by category for new studies*

| **Study** | **Adequate sequence generation?** | **Allocation concealment?** | **Incomplete outcome data addressed?** | **Free of selective reporting?** | **Blinding (participant)** | **Blinding (personnel)** | **Blinding (assessor)** |
| --- | --- | --- | --- | --- | --- | --- | --- |
| Barber et al (2016) | 1 | 1 | 1 | 0 | 2 | 2 | 2 |
| Carey et al (2015) | 1 | 1 | 0 | 1 | 2 | 2 | 2 |
| Conner et al (2017) | 0 | 2 | 0 | 0 | 2 | 0 | 0 |
| McCambridge et al (2013) | 0 | 0 | 0 | 0 | 0 | 0 | 0 |
| McDermott et al (2018) | 0 | 0 | 0 | 0 | 0 | 0 | 0 |
| Meier et al (2017) | 0 | 1 | 2 | 0 | 2 | 0 | 2 |
| O’Carroll et al (2015) | 0 | 0 | 0 | 0 | 1 | 0 | 0 |
| O’Carroll et al (2016) | 1 | 0 | 0 | 0 | 0 | 0 | 0 |
| Wilding et al (2018) | 0 | 1 | 2 | 1 | 0 | 0 | 1 |
| Wood et al (2014) | 2 | 2 | 1 | 1 | 2 | 2 | 2 |
